# A Novel TetR-Regulating Peptide Turns off rtTA-Mediated Activation of Gene Expression

**DOI:** 10.1371/journal.pone.0096546

**Published:** 2014-05-08

**Authors:** Sebastian Schmidt, Christian Berens, Marcus Klotzsche

**Affiliations:** Lehrstuhl für Mikrobiologie, Department Biologie, Friedrich-Alexander-Universität Erlangen-Nürnberg, Erlangen, Germany; Charité-University Medicine Berlin, Germany

## Abstract

Conditional regulation of gene expression is a powerful and indispensable method for analyzing gene function. The “Tet-On” system is a tool widely used for that purpose. Here, the transregulator rtTA mediates expression of a gene of interest after addition of the small molecule effector doxycycline. Although very effective in rapidly turning on gene expression, the system is hampered by the long half-life of doxycycline which makes shutting down gene expression rapidly very difficult to achieve. We isolated an rtTA-binding peptide by *in vivo* selection that acts as a doxycycline antagonist and leads to rapid and efficient shut down of rtTA-mediated reporter gene expression in a human cell line. This peptide represents the basis for novel effector molecules which complement the “Tet-system” by enabling the investigator to rapidly turn gene expression not just on at will, but now also off.

## Introduction

The official completion of the human genome project in 2003 was perceived as a landmark event in biological science [1], [2]. However, to make the most of the vast amount of sequence data, several questions have to be addressed, i.e. what fraction of the genome is functional or which of these genes are of therapeutical interest [3], [4]. One powerful and indispensable means to analyze gene function is conditional regulation of gene expression and, consequently, many systems have been developed for this purpose [5]–[9]. A widely used approach is based on tetracycline-dependent gene regulation which originated from the bacterial transcription factor Tet Repressor (TetR) in combination with a TetR-responsive promoter [10]. Tetracycline (tc) derivatives are then used as small molecule effectors to efficiently regulate the expression of the cloned gene of interest. These TetR-based regulatory systems have been frequently used in a variety of different organism ranging from bacteria to mammals [11]–[15]. For a successful application in eukaryotic organisms, TetR has to be modified by adding regulatory domains like the VP16 activation domain derived from Herpes Simplex virus as a fusion to the C-terminus of TetR [16]. The reverse tc-dependent transactivator rtTA2^S^-M2, which is composed of a reverse TetR variant and a VP16-derived minimal activation domain [17], is a highly efficient representative of these so-called tc-dependent transregulators. Addition of the tc derivative doxycycline (dox) leads to binding of this rtTA variant to the TetR-responsive promoter and to subsequent activation of gene expression. This “Tet-On” system is used efficiently to switch on gene expression and analyze the resulting effects [18]–[21]. However, due to the long half-life of dox [22], switching off target gene expression can only be achieved by replacing the dox-containing medium with dox-free medium, or, in animal studies, to supply dox-free drinking water or food. In both cases, this leads only to a moderate and slow decrease in target gene expression [23]–[25]. If a controlled and rapid shut down of target gene expression is necessary, small molecule effectors that act as dox antagonists would be of great benefit.

To date, only a single small molecule has been isolated that acted as an antagonist of the tc-dependent transactivator tTA in bacteria [26]. However, this publication was never followed-up by any additional studies, despite strong interest in such a molecule to rapidly switch target gene expression on and off *in vivo*.

Besides tc derivatives, peptides have recently been isolated as novel effectors for TetR. The first to be discovered was called TetR Inducing Peptide (TIP) and induces the TetR variant TetR(B) in bacteria like tc [27]. X-ray structural analysis revealed partial binding of TIP inside the tc-binding pocket of TetR [28]. TetR regulating peptides are mostly composed of 12 to 16 amino acids and can exert highly diverse effects such as induction (TIP, “TetR inducing peptide”), anti-induction (TAP, “TetR anti-inducing peptide”) and co-repression (TCP, “TetR co-repressing peptide”) [29]. The regulatory flexibility displayed by these peptides, together with the lack of small molecule antagonists, led us to attempt to isolate regulatory peptides for the efficient and most widely used transregulator rtTA2^S^-M2. During the experimental procedure, a peptide was discovered that acted as a dox antagonist in both bacteria and a human cell line. Extensive analysis of the peptide revealed residues which are necessary for its antagonistic activity and shed light on its potential binding site on rtTA. We could further show that expression of the peptide in a human cell line led to a significantly faster and stronger decline in rtTA-mediated activation of gene expression compared to samples in which dox was removed by medium exchange. Therefore, this novel rtTA-regulating peptide represents the basis for developing small molecule antagonists that could complement the widely used Tet-On system with an efficient means to rapidly switch off rtTA-mediated activation of target gene expression at will.

## Results and Discussion

### Selecting rtTA-binding peptides

**Figure 1:**
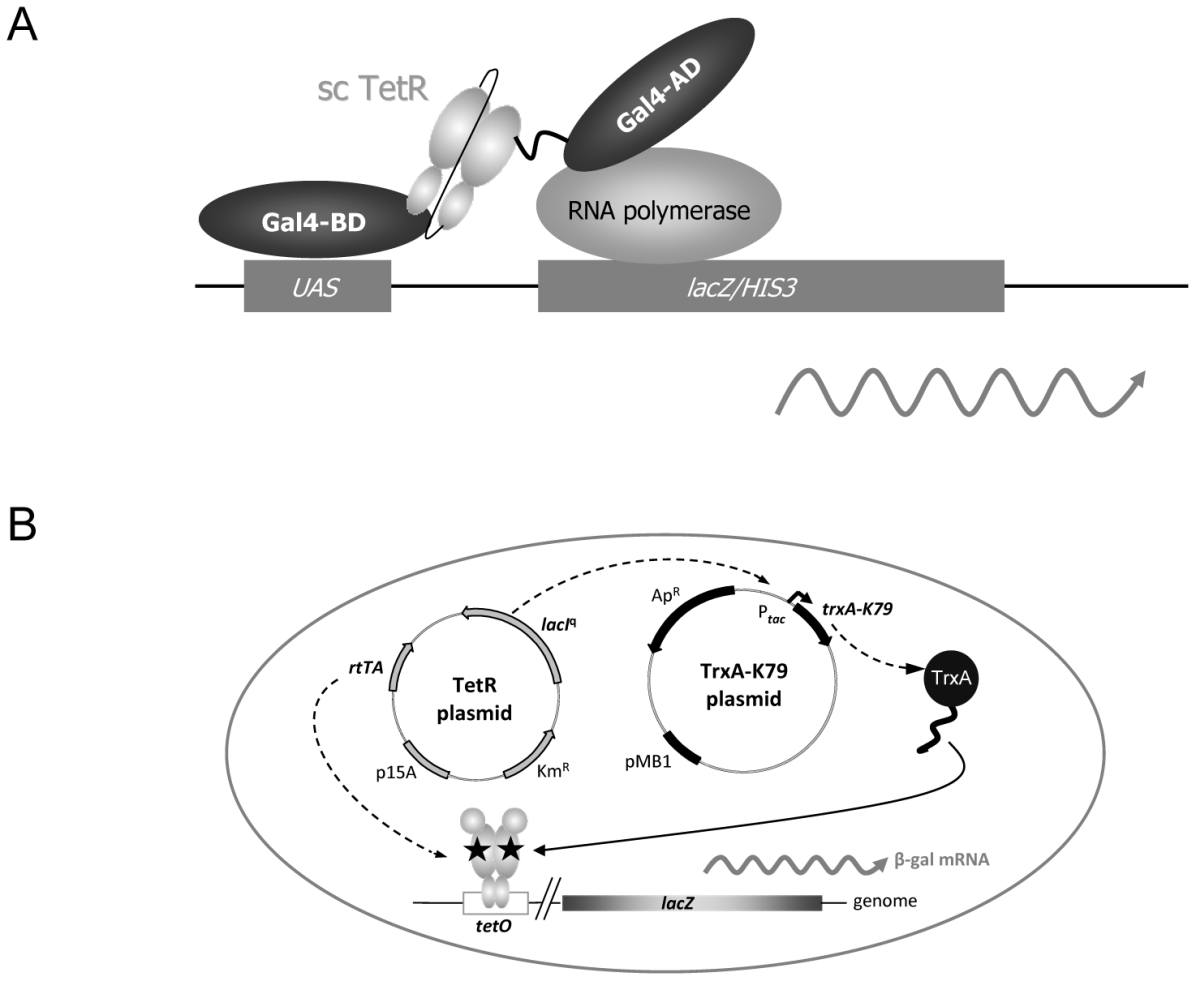
Isolation and *in vivo* analysis of the peptide K79. (**A**) Scheme of the Yeast Two Hybrid selection used to isolate rtTA-binding peptides. The yeast strain harbors a *lacZ* reporter gene and a HIS3 selection marker controlled by an upstream activating sequence (UAS). The TetR moiety of rtTA was used as a single-chain variant (sc TetR) fused to the C-terminus of the Gal4 DNA binding domain (Gal4-BD). A peptide library was fused to the C-terminus of the Gal4 activation domain (Gal4-AD). (**B**) *E. coli* WH207(λ*tet*50) containing *lacZ* under Tet control was used to analyze K79-mediated effects in *E. coli*. The TetR expressing plasmid constitutively expresses LacI and the respective TetR variant. The TetR dimer, depicted by grey ovals indicating the DNA- and inducer-binding domains, controls transcription of *lacZ*. The TrxA-K79 expressing plasmids encode either wildtype TrxA or the C-terminal K79 fusion to TrxA under control of the *P*_tac_ promoter. Antagonistic activity of the TrxA-K79 peptide fusion leads to an increase of β-galactosidase expression by reducing dox-mediated repression of the reverse TetR variant. Dox is shown as black pentagon.

RtTA-binding peptides were selected using a Yeast Two Hybrid approach illustrated in Figure 1A. A plasmid was constructed encoding a single-chain variant of the reverse transregulator rtTA2^S^-M2 [17] fused to the C-terminus of the Gal4 DNA binding domain. This regulator is composed of the reverse TetR variant TetR(B)S12G-E19G-A56P-D148E-H179R fused to a VP16-derived minimal activation domain [17]. In order to prevent the activation of reporter gene expression by the rtTA, this domain was deleted in the variant used during the selection. This “bait protein” encoding plasmid was designated pGBT9-sc(sM2Δ) and contains a *trp1* selection marker. A second plasmid was used which encodes a randomized peptide library fused to the C-terminus of the Gal4 activation domain. The library consisted of approximately 10^7^ randomized 16-mer peptides (Matchmaker™, Clontech). This “prey protein” encoding plasmid was designated pGAD-GH and features a leu2 selection marker. Both plasmids were used to transform the yeast strain AH109 which is characterized by a his3 auxotrophy marker and a Gal4 binding site (upstream activating sequence (UAS)) cloned upstream of a lacZ/his3 reporter gene. The selection procedure was carried out on media lacking tryptophan, leucine and histidine. By carrying out multiple independent rounds of selection we ensured that the complexity of the peptide library was covered as calculated from the efficiency of transformation.

### *In vivo* analysis of rtTA-binding peptides in *E. coli*

**Figure 2:**
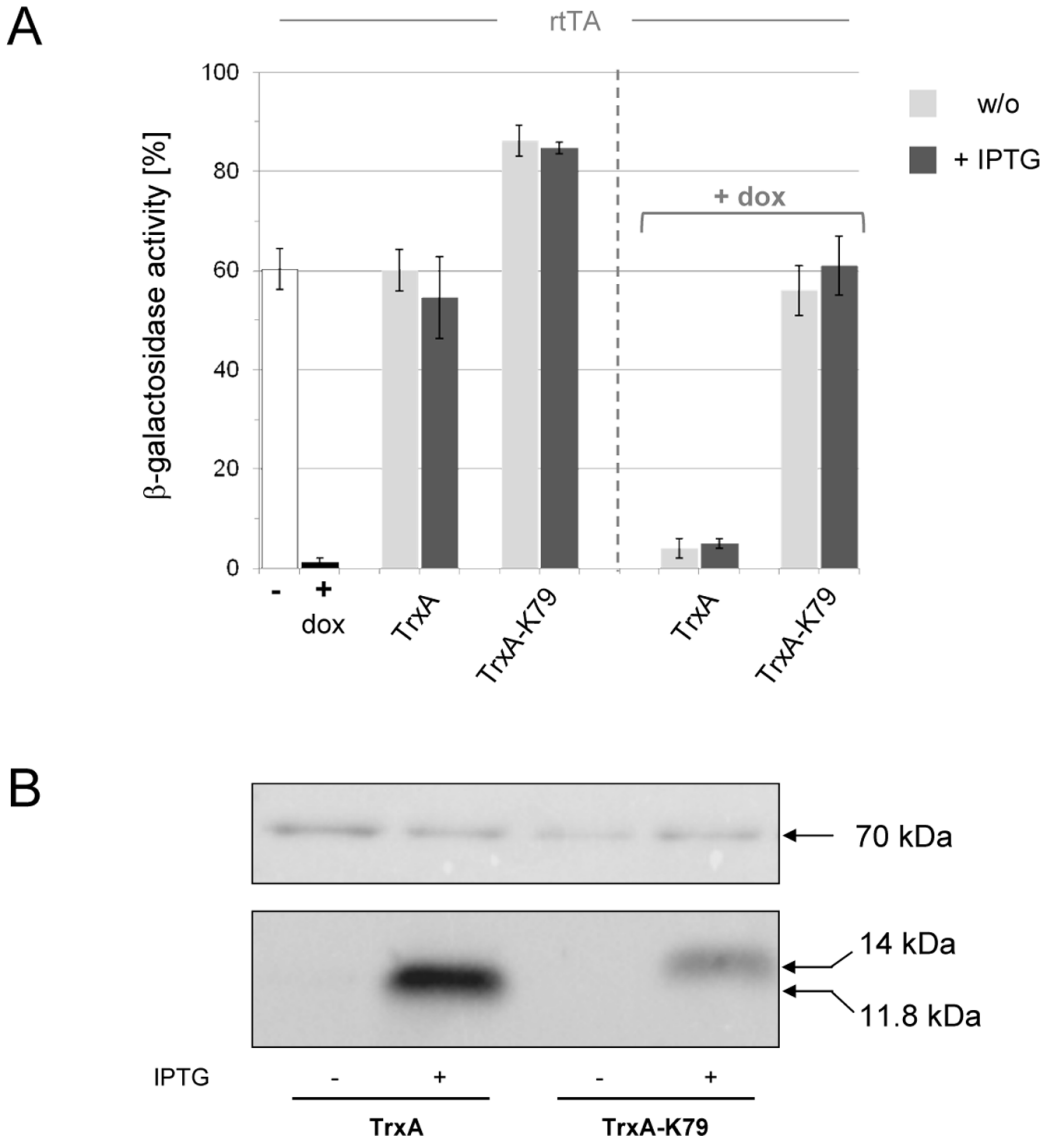
Antagonistic activity of K79 in *E. coli*. (**A**) K79-mediated effects were analyzed using the *in vivo* system depicted in Figure 1 in which the reverse TetR variant rtTA controls expression of a *lacZ* reporter gene. In the absence of dox (white bar), β-galactosidase activity is maximal, while it is reduced by rtTA-mediated repression of *lacZ* expression in the presence of dox (black bar). Expression of TrxA has no effect on β-galactosidae activity, while expression of the C-terminal peptide fusion TrxA-K79 leads to an increase in β-galactosidae activity. Basal expression of TrxA and TrxA-K79 is shown as light grey bar, IPTG-induced overexpression as dark grey bar. Expression of TrxA in the presence of dox (right-hand side of the diagram) has no effect on β-galactosidae activity. Expression of TrxA-K79 in the presence of dox leads to a complete reversal of rtTA-mediated repression of *lacZ*. The data are shown as mean values ± standard deviations. (**B**) Western blot analysis of TrxA and TrxA-K79 expression levels in *E. coli* using an anti-TrxA antibody (bottom row). Protein lysates were obtained from the log-phase cultures used in (A). IPTG was used at 60 µM when indicated. DnaK, which served as loading control, was detected with an anti-DnaK antibody (top row). Molecular weights are indicated on the right-hand side. TrxA, 11.8 kDa; TrxA-K79, 14 kDa; DnaK, 70 kDa.

Clones showing a positive phenotype in the LacZ filter assays were chosen for further characterization of their ability to regulate rtTA. For the *in vivo* analysis, an *E. coli* strain harboring a TetR-controlled *lacZ* reporter gene was used (Figure 1B). Peptide sequences were determined, PCR-amplified and cloned as C-terminal fusions to thioredoxin A (TrxA) which acted as a protein scaffold to stably express the selected peptides [27]. TrxA-peptide fusions were under control of *P_tac_*, hence, their expression is induced upon addition of IPTG. rtTA, on the other hand, was constitutively expressed and, in the presence of the tetracycline derivative doxycycline (dox), repressed transcription of the *lacZ* reporter gene approximately 20-fold (Figure 2). The analysis of 19 LacZ-positive clones revealed that none acted like a dox surrogate which would have resulted in co-repression of *lacZ* expression in *E. coli*. However, one candidate – designated K79 – led to an increase in reporter gene expression which could be interpreted as K79 binding triggering a decrease in residual DNA-binding activity of the reverse TetR variant (Figure 2).

In order to further characterize this phenomenon, we expressed TrxA-K79 in the presence of dox. As depicted in Figure 2, expression of TrxA-K79 completely abolished dox-mediated repression of *lacZ* expression. Interestingly, this was also observed in the absence of IPTG, when only basal expression of TrxA-K79 occurs due to the leakiness of the *P_tac_* promoter – an indication of the peptide's high activity. Furthermore, we determined that the antagonistic effect is peptide-specific, because it was not observed when TrxA was expressed without peptide fusion (Figure 2). Hence, K79 behaved like a dox antagonist in *E. coli*. Western blot analysis using an anti-TrxA antibody further revealed that TrxA-K79 was expressed, even though at lower steady state levels than wildtype TrxA (Figure 2B).

### Mutational analysis of the peptide K79

**Figure 3:**
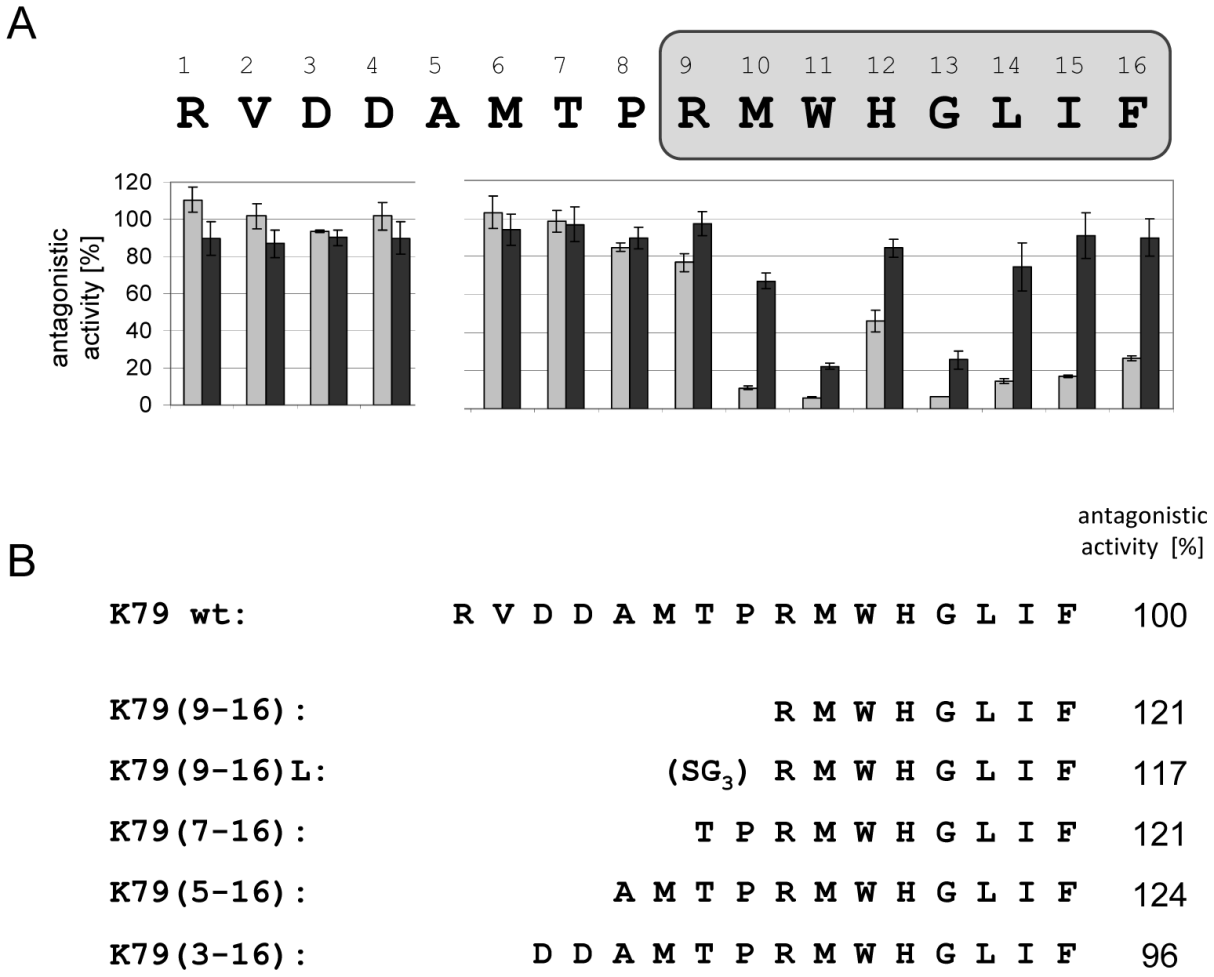
Sequence requirements necessary for the antagonistic activity of K79. (**A**) Alanine scanning was done in order to identify residues within the K79 peptide sequence contributing to its antagonistic activity. Each position was mutated to alanine and the respective TrxA-K79 fusion was analyzed in *E. coli* as depicted in Figure 1. The K79 sequence is shown with one-letter abbreviations and the effect of each alanine exchange on rtTA-controlled *lacZ* expression is depicted underneath the sequence in % antagonistic activity normalized to K79 wildtype which was set to 100%. (**B**) K79 variants, in which the N-terminal residues 1 to 9 were truncated successively, were analyzed. The antagonistic activity of the respective K79 deletion variant is shown on the right-hand side of the sequence. The antagonistic activity of the K79 wildtype was set to 100%.

**Figure 4:**
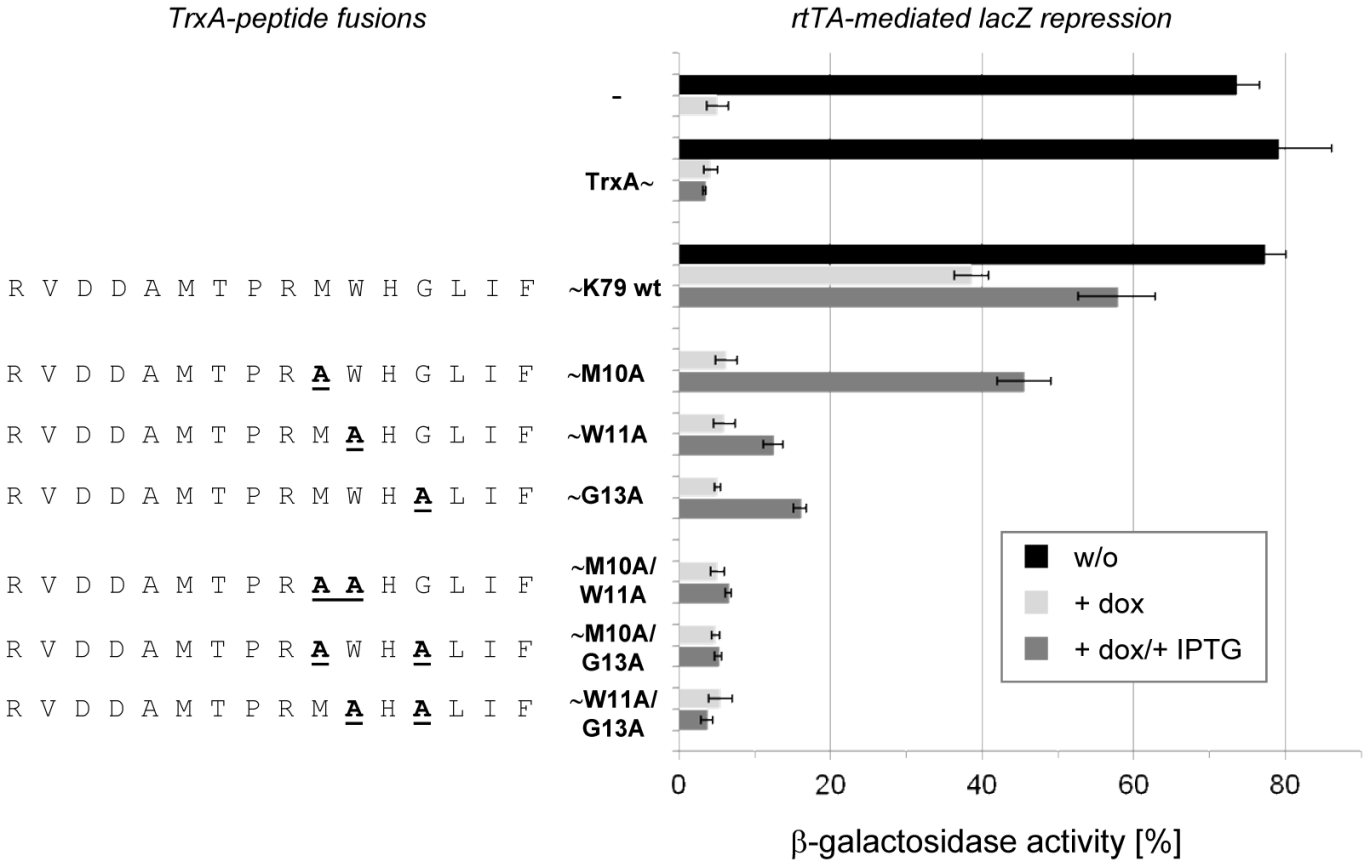
Analysis of K79 “loss of function” mutants in *E. coli*. K79 mutants were expressed as C-terminal TrxA fusions and analyzed using the *in vivo* system shown in Figure 1. Mutations M10A, W11A and G13A were combined to obtain the double mutants M10A/W11A, M10A/G13A and W11A/G13A. Their ability to abolish rtTA-mediated repression of *lacZ* is compared to K79 wildtype. β-galactosidae activity in the absence of dox is depicted with black bars. Basal expression of TrxA or a specific TrxA-K79 fusion in the presence of dox is depicted with white bars, while IPTG-induced overepression is depicted with grey bars. The sequences of the K79 mutants are shown with one-letter abbreviation on the left-hand side. The data are shown as mean values ± standard deviations.

To analyze the contribution of each individual peptide residue to the antagonistic activity, we performed an alanine scan in which alanine exchange mutants were constructed as C-terminal TrxA fusions (Figure 3A). These K79 variants were then analyzed in the *E. coli* screening system and their activities monitored via *lacZ* expression. Interestingly, exchanges at positions 1 to 8 were completely tolerated, since these mutants were as active as the K79 wildtype. The K79 mutants R9A, H12A, L14A, I15A and F16A showed only a minor decrease in their activities while mutants M10A, W11A and G13A were more strongly impaired in their ability to regulate rtTA-controlled gene expression. When these single-site mutations were combined by constructing the double mutants M10A-W11A, M10A-G13A and W11A-G13A, the activity of K79 was completely lost (Figure 4). Hence, cooperative action of these residues is necessary for the antagonistic activity of the peptide. A feasible conclusion would be that G13, due to the lack of a functional chemical group, plays a crucial part in permitting flexibility of K79 while adopting its active conformation. In contrast, M10 and W11 might be important by forming strong interactions with rtTA via their functional groups. A similar result was found for the TetR inducing peptides TIP and TIP2, since the crystal structures of both, [TetR⋅TIP] and [TetR⋅TIP2], revealed tryptophan residues localized within the tc-binding pocket forming crucial interactions to residues of TetR [28], [30]. For both peptides, the activity was lost when the tryptophan residues were exchanged to alanine [29], [31].

### Analysis of truncated K79 variants

Since the outcome of the alanine scanning analysis suggested no significant contribution of residues 1 to 8 to the activity of K79, we addressed the question whether truncated versions of the peptide might be active as well. For that purpose we constructed five K79 variants in which the N-terminal residues were successively deleted (see Figure 3B). All of these variants, even the shortest 8-mer peptide K79(9–16), were as active as K79 wildtype corroborating the aforementioned assumption. In summary, only the C-terminal residues 9 to 16 of K79, in particular M10, W11 and G13 contribute to the antagonistic activity of K79, but not residues 1 to 8 which are flanked N-terminally by TrxA and C-terminally by the activity-mediating residues 9 to 16. A similar behavior was observed with TetR-regulating peptides TIP2 and TAP1, whose N-terminal residues are permissive to alanine exchanges, while their C-terminal residues are not [29]. Since the crystal structures of [TetR⋅TIP], [TetR⋅TIP2] and [TetR⋅TAP1] complexes revealed at least partial binding of the peptide inside the tc-binding pocket [28], [30], a feasible conclusion is that binding of K79 residues 9 to 16 also occurs inside the rtTA effector binding pocket.

### Regulation of wildtype TetR by K79

**Figure 5:**
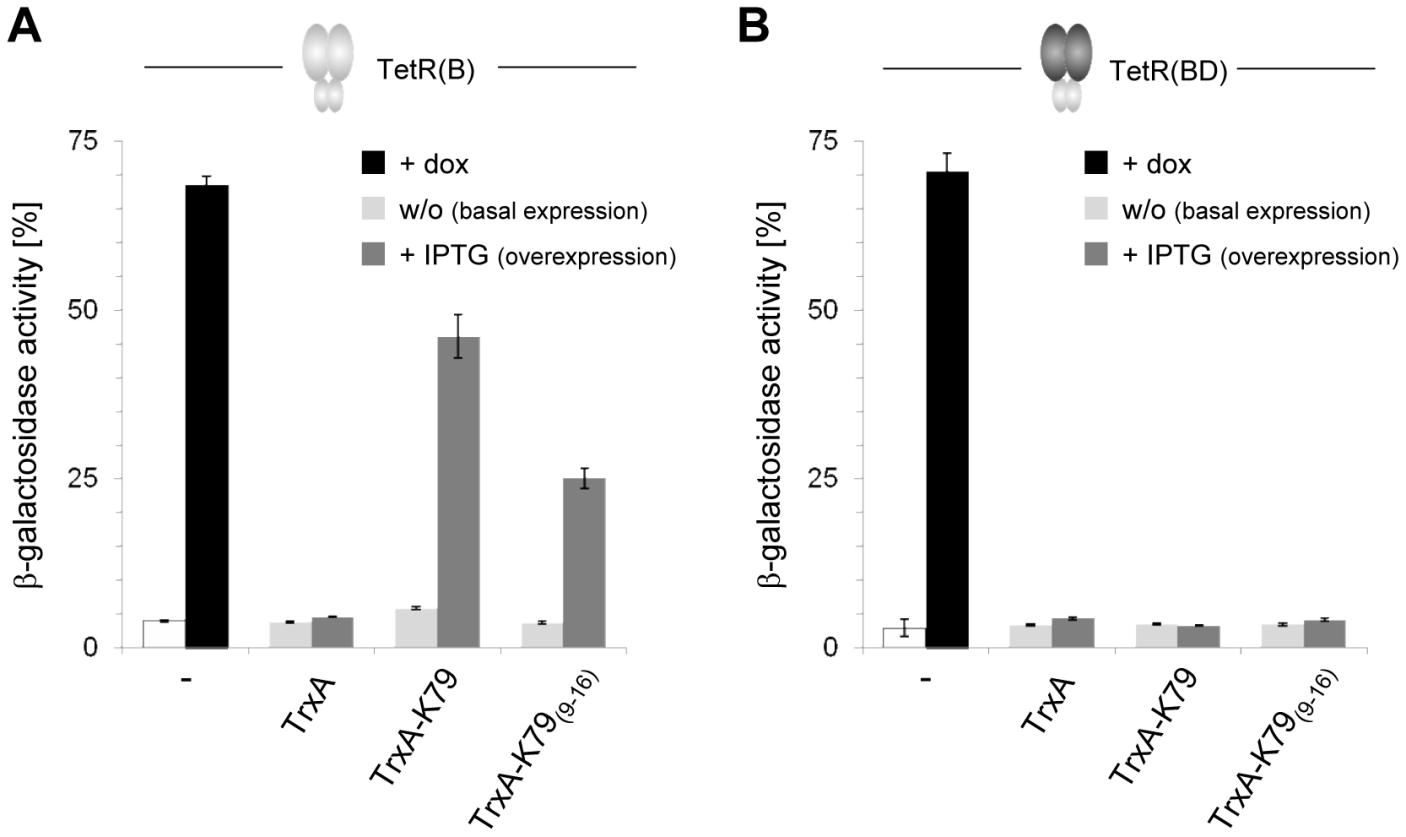
K79-mediated effects on gene expression controlled by wildtype TetR. (**A**) K79 variants were expressed in an *E. coli* strain in which *lacZ* expression is controlled by TetR(B) wildtype. In the absence of dox (white bar), *lacZ* is repressed by binding of TetR to *tetO* (compare Figure 1), while in the presence of dox (black bar), *lacZ* expression is induced. Basal expression of TrxA or a specific TrxA-K79 fusion is depicted with light grey bars, and IPTG-induced overepression is depicted with dark grey bars. The data are shown as mean values ± standard deviations. (**B**) Same as in (A), but *lacZ* expression is controlled by the wildtype TetR variant TetR(BD) in which the inducer binding-/dimerization domain is replaced against the class D sequence.

Since the residues comprising the tc-binding pocket are identical in rtTA and wildtype TetR(B), we addressed the question whether K79 could also regulate wildtype TetR(B). Therefore, the rtTA-expressing plasmid was exchanged for a TetR(B)-expressing plasmid in the *in vivo E. coli* assay. K79 was again expressed as C-terminal fusion to TrxA in the absence and presence of 60 µM IPTG. Samples to which dox was added were also included to address the antagonistic activity of K79 which, in case of wildtype TetR(B), would lead to a decrease in dox-mediated induction of reporter gene expression. Interestingly, K79 behaved similar to the peptide-based inducers TIP and TIP2 and no antagonistic activity was detected. In the presence of IPTG, expression of TrxA-K79 led to 60% β-galactosidase expression as shown in Figure 5A. Addition of dox in the presence of TrxA-K79 yielded no significant effect compared to the samples not treated with dox, hence, no antagonistic activity was observed (data not shown). Therefore, in *E. coli*, K79 behaves like an anti-corepressing peptide for rtTA, but like an inducer for TetR(B). The TetR variant TetR(BD) was also included in this analysis. In TetR(BD), the inducer-binding and dimerization domain, comprised of helices α4 to α10, is replaced by the TetR class D sequence, hence, only the DNA binding domain (α1 to α3) is identical to TetR(B). TetR(BD) was not regulated by TrxA-K79 (Figure 5B). Based on this result, it can be excluded that K79 regulates TetR via binding to its DNA binding domain.

### Characterization of K79 binding inside the tc-binding pocket

**Figure 6:**
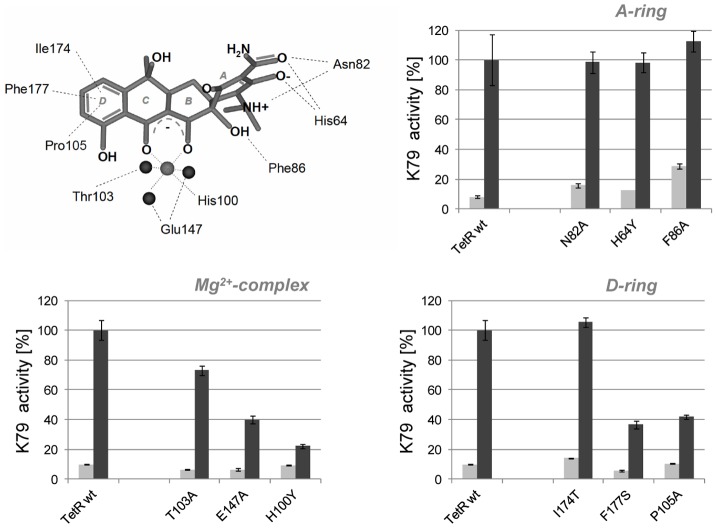
Structure-function analysis of K79 activity and inducer binding pocket of TetR. K79 activity is monitored in *E. coli* in which *lacZ* expression is controlled by TetR(B) wildtype and various TetR(B) mutants with single-residue exchanges located within the inducer-binding pocket. Mutated amino acids are depicted with respect to their orientation towards tetracycline (tc). The magnesium ion within the tc⋅Mg^2+^ complex is illustrated as light grey ball, while water molecules are shown as dark grey balls. TetR mutants are grouped with regard to their tc contact (A-ring, Mg^2+^-complex and D-ring contacting residues). K79 activity with TetR(B) wildtype was set to 100% β-galactosidae activity. Basal expression of TrxA-K79 is depicted with light grey bars, and IPTG-induced overepression is depicted with dark grey bars. The data are shown as mean values ± standard deviations. Values of the β-galactosidae activities monitored are also listed in the Table 1.

To further corroborate the assumption that binding of K79 occurs inside the tc-binding pocket of TetR, mutants with single amino acid exchanges in this region were investigated. We first analyzed mutants with exchanges in the so-called anchor residues H64, N82 and F86 that contact the A-ring of tc (compare Figure 6). These residues are crucial for tc recognition and exchanges generally lead to non-tc-inducible TetR mutants. Interestingly, the variants H64Y, N82A and F86A were fully inducible by TrxA-K79 (Figure 6), similar to results obtained with TIP [27], but not with TIP2 [29] (compare Table 1). Identical behavior of TIP and K79 was also found for mutants with exchanges in residues involved in Mg^2+^-complexation. While only a moderate effect was observed for T103A, inducibility of the mutants H100Y and E147A was strongly impaired. Next, we analyzed residues that form the hydrophobic pocket, for example, P105, I174 and F177 which contact the D-ring of tc (Figure 6). The inducibility of the mutants P105A and F177S was decreased to about 40%, while no effect was observed for the mutant I174T. Concerning these mutants, no concordant behavior is seen for K79, TIP and TIP2.

**Table 1:** Activity of TetR-inducing peptides K79, TIP and TIP2 to induce TetR variants compared to TetR wildtype.

TetR mutant	TetR-inducing peptide*		
	K79	TIP**	TIP2***
N82A	**+ (99±7)**	**<**	**<**
F86A	**+ (113±9)**	**+**	**<**
H64Y	**+ (98±7)**	**+**	**−**
H100Y	**− (22±2)**	**−**	**−**
T103A	**< (73±3)**	**<**	**+**
E147A	**< (40±3)**	**<**	**<**
P105A	**< (42±5)**	**−**	**+**
I174T	**+ (105±13)**	**−**	**+**
F177S	**< (37±4)**	**+**	**+**
G102R	**< (60±4)**	**−**	**−**

*Inducibility of denoted TetR variants was measured in a β-galactosidase assay. Inducibility of TetR wildtype was set to 100% activity.

**+**: activity >75%; **<**: activity <75%; −: activity <25%.

**data from [27].

***data from [29].

Although a clear consensus can be found in the behavior of K79 and TIP with regard to A-ring contacting residues and residues involved in Mg^2+^-complexation, the overall recognition pattern of K79 differs strongly from that determined for TIP2. Interestingly, a consensus in the behavior of all three peptides is observed only with the mutants H100Y and G102R, which are both involved in the allosteric transition of TetR triggered by inducer binding [32]. In summary, the data clearly indicate that binding of K79 occurs at least partially inside the tc-binding pocket and with a recognition pattern similar to TIP.

### Functionality of K79 in mammalian cell lines

**Figure 7:**
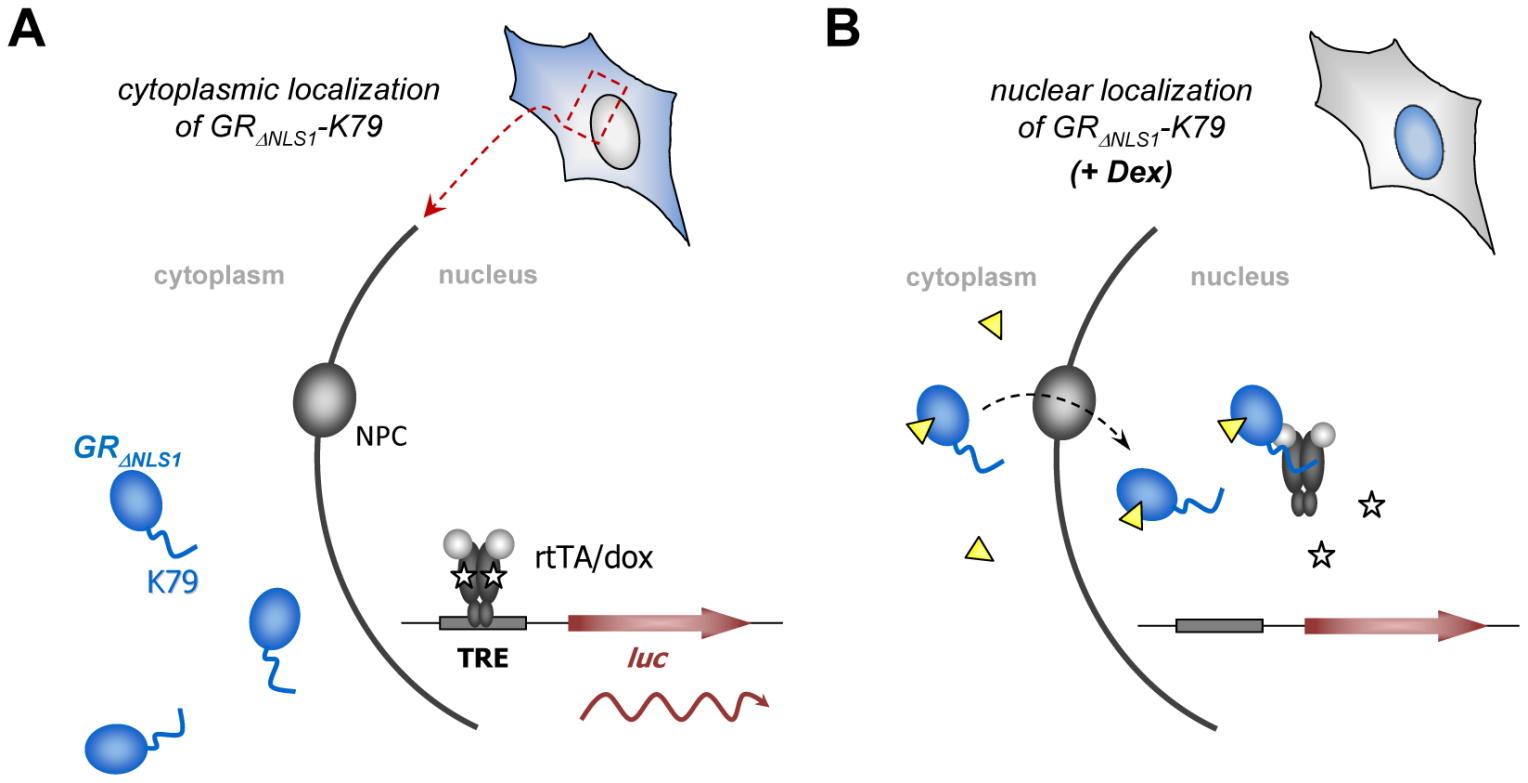
Methodology to characterize K79 activity in HeLa cells. (**A**) The HeLa cell line harbors a stably integrated luciferase gene (*luc*) under control of a Tet-responsive promoter (TRE). In the presence of dox (white pentagon), the reverse transregulator rtTA binds to the TRE and leads to activation of gene expression. The peptide K79 is fused to the C-terminus of a glucocorticoid receptor variant (GR_ΔNLS1_), and, in the absence of dexamethasone (yellow triangle), resides within the cytoplasm where it cannot interact with nuclear rtTA. (**B**) After addition of dexamethasone (Dex), GR_ΔNLS1_-K79 is transported to the nucleoplasm via the nuclear pore complex (NPC) where it can interact with rtTA via the K79 moiety. Due to the antagonistic activity of K79, this leads to a decrease in *luc* expression.

**Figure 8:**
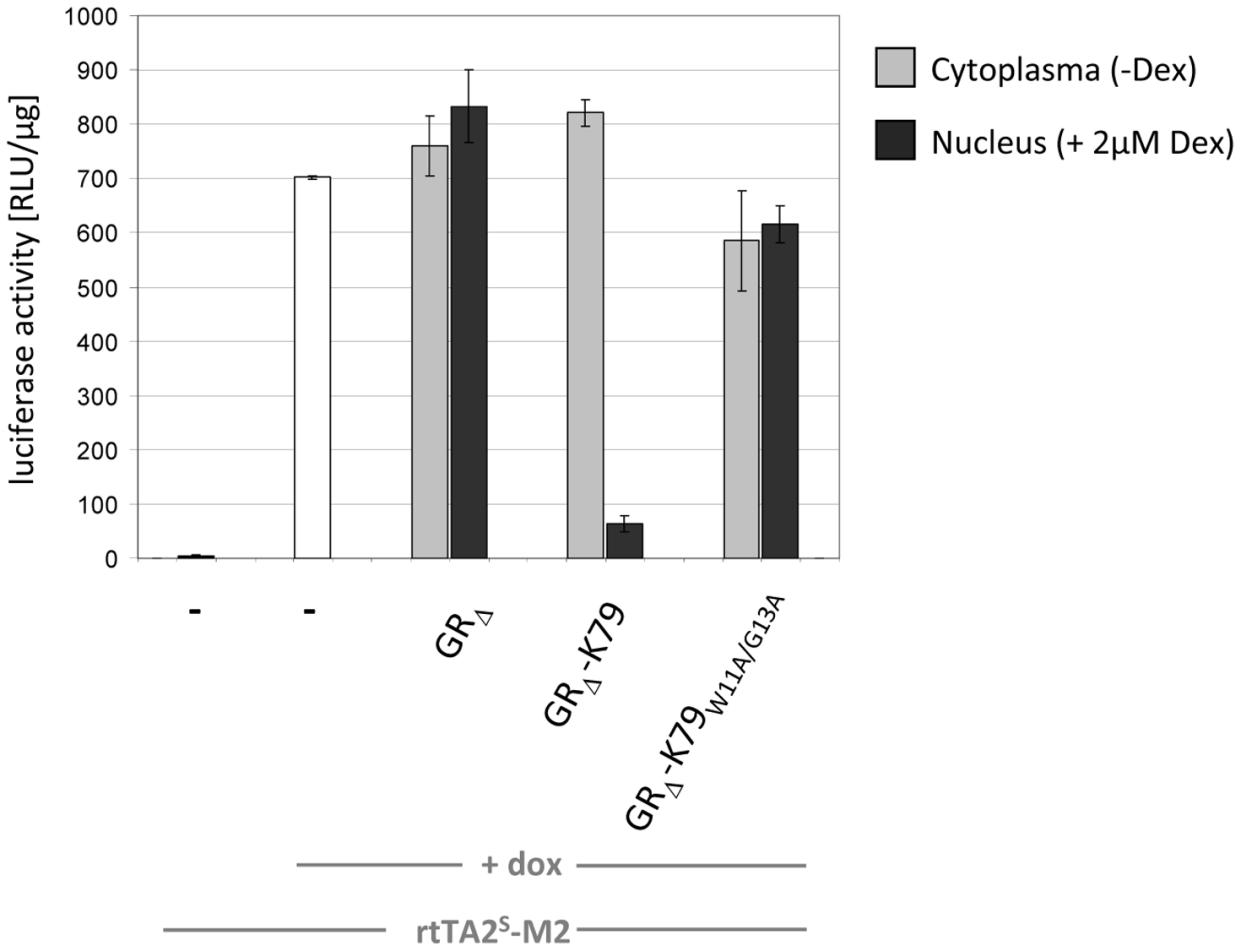
*In vivo* characterization of K79 activity in HeLa cells. Analysis of K79-mediated regulation of *luc* expression in a HeLa cell line as described in Figure 7. Luciferase expression is controlled by rtTA and, in the presence of doxycycline (dox), leads to activation of gene expression (white bar). K79 is expressed as C-terminal fusion to a glucocorticoid receptor variant (GR_ΔNLS1_). In the absence of dexamethasone, GR_ΔNLS1_ and GR_ΔNLS1_-K79 reside within the cytoplasm (grey bars), while in the presence of dexamethasone (Dex), GR_ΔNLS1_ and GR_ΔNLS1_-K79 are transported to the nucleus (black bars). When dox is used in the latter samples, GR_ΔNLS1_-K79 can interact with rtTA via the K79 peptide which leads to reversal of rtTA-mediated activation of *luc* expression. The data are shown as mean values ± standard deviations.

After having analyzed the behavior of K79 in *E. coli*, we addressed the question whether the peptide is also functional in mammalian cell lines. This would open up the fascinating possibility of using K79 as a dox antagonist to efficiently and rapidly turn off rtTA-mediated gene expression. The human cell line we used in this study is designated HeLa HLF33 [33] and is characterized by a stably integrated luciferase reporter gene (*luc*) as shown in Figure 7. TetR-controlled *luc* expression is achieved by a TetR-responsive element containing seven consecutive *tetO_2_* boxes and a Cytomegalovirus minimal promoter [16]. The rtTA-encoding plasmid pWHE120(sM2), in which expression of the protein is driven by a constitutive strong Cytomegalovirus promoter/enhancer, was used in transient transfection experiments to determine the regulatory properties of the system. In the absence of dox, only basal expression of *luc* occurs. After addition of 2 µM dox, rtTA binds to the Tet-responsive element thereby activating *luc* expression via its minimal activation domain. This led to an 1800-fold increase in luciferase activity (Figure 8). In order to analyze K79 in this setup, we took advantage of a method we previously published [34]. In short, peptides to be analyzed *in vivo* are fused to a glucocorticoid receptor variant in which the nuclear localization signal 1 is inactivated (GR_ΔNLS1_) [35]. In the absence of glucocorticoids or artificial derivatives thereof, like dexamethasone, GR_ΔNLS1_ resides within the cytoplasm. Hence, peptides fused to GR_ΔNLS1_ cannot interact with the TetR-based transcription factor. After addition of dexamethasone, GR_ΔNLS1_ shuttles into the nucleus permitting the interaction of a fused peptide with the TetR-based regulator, thereby altering the expression of the *luc* reporter gene (compare Figure 7). Hence, a plasmid was constructed that encodes a C-terminal GR_ΔNLS1_-K79 fusion and transient transfections were performed using the aforementioned cell line in which *luc* expression is controlled by rtTA. Transfection of a control plasmid encoding GR_ΔNLS1_ without a peptide fusion had no effect on *luc* expression (Figure 8). However, when a plasmid encoding the GR_ΔNLS1_-K79 fusion was used, a 10-fold decrease in *luc* expression was observed. This clearly demonstrates the antagonistic activity of K79 in a setup in which gene expression is activated by rtTA. As an additional control, we constructed a plasmid encoding a GR fusion to the K79 variant K79-W11A-G13A. These mutations completely abolish K79 activity in *E. coli* (Figure 4). Transient transfection of this variant did not affect reporter gene expression, corroborating the specific K79-mediated effect on rtTA-controlled regulation of gene expression (Figure 8).

### K79 acts as a dox antagonist

**Figure 9:**
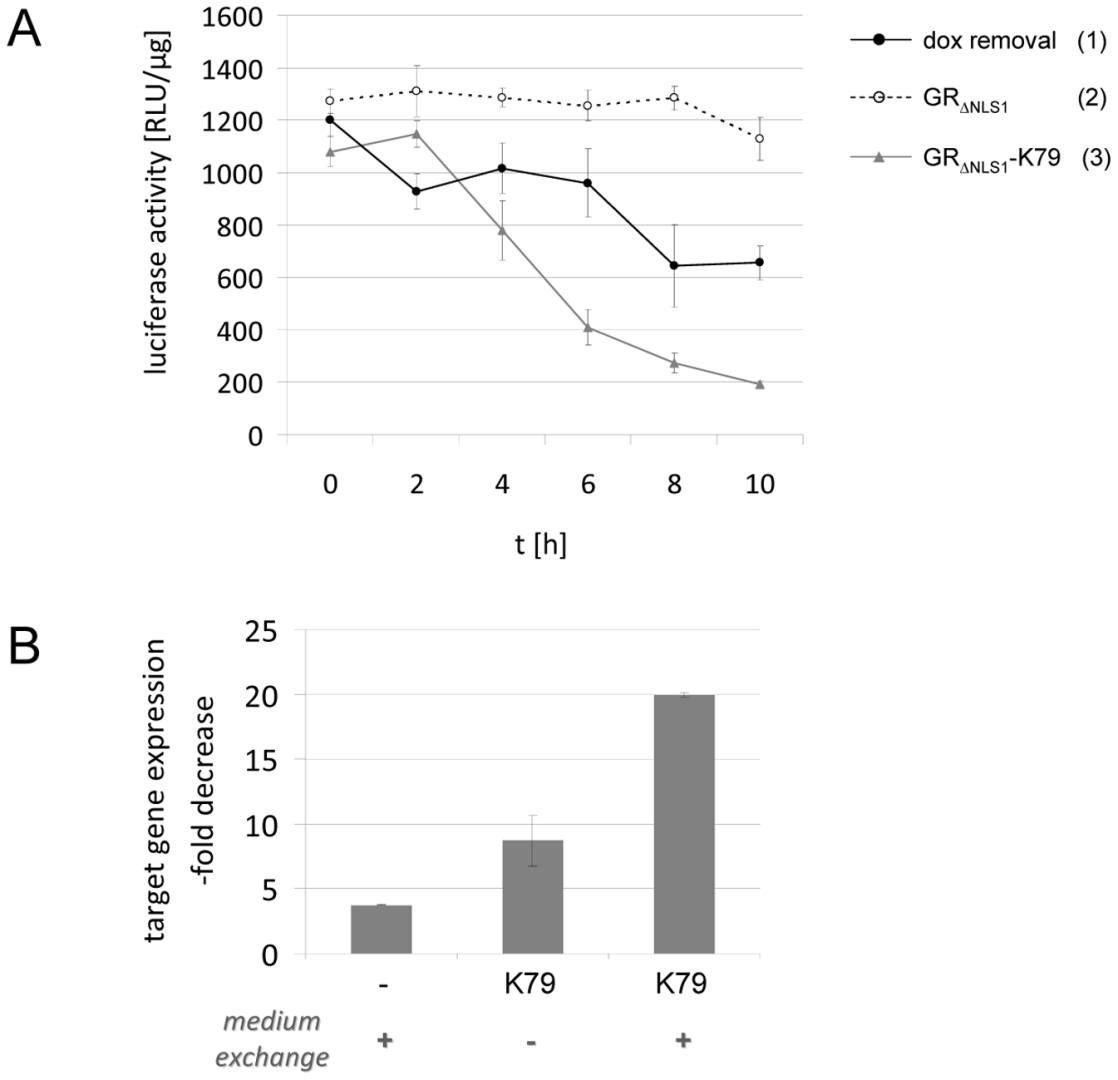
Switching off gene expression: efficiency of K79 compared to dox removal. (**A**) The HeLa cell line illustrated in Figure 7 was transiently transfected with an rtTA-encoding plasmid – either alone, or double-transfected with a GR_ΔNLS1_-K79-, or a GR_ΔNLS1_-encoding plasmid, respectively. Before measuring luciferase activity, dox-containing medium was exchanged with dox-free medium at the indicated time points for cells transfected with rtTA only (sample 1; black circles, solid line). For cells additionally transfected with GR_ΔNLS1_ (sample 2; open circles, broken line) or GR_ΔNLS1_-K79 (sample 3; grey triangles, solid line), respectively, 2 µM dexamethasone was added at these time points. The data are shown as mean values ± standard deviations. (**B**) Experimental procedure as in (A). 10 h time points are shown as x-fold decrease in luciferase expression. The diagram illustrates the effect of dox-removal by medium exchange (left bar), GR_ΔNLS1_-K79 expression (middle bar) and GR_ΔNLS1_-K79 expression together with dox-removal by medium exchange (right bar).

A potential application of K79 would be its use as a dox antagonist to switch off rtTA-mediated activation of gene expression at will. To date, only a single small molecule has been isolated as an antagonistic effector of the TetR-containing synthetic eukaryotic transcription factor tTA [26]. Unfortunately, this publication was never followed-up by any additional data, despite strong interest in such molecules. Hence, up to now, turning off rtTA-mediated activation of target gene expression can only be achieved by exchanging the dox-containing cell culture medium with dox-free medium, or, in the case of animal studies by providing dox-free drinking water or food. Using this procedure, the dox concentration is only slowly decreased due to its long half-life [22] and due to the fact that medium exchange does not affect dox within or attached to the cells. Furthermore, dox molecules bound to rtTA still lead to rtTA-mediated activation of gene expression. For example, by generating transgenic mice in which Ras expression – titratable in a dox-dependent manner – is controlled by a Tet-responsive promoter and rtTA, Sarkisian et al. showed that Ras expression and Ras pathway activation returned to levels comparable to those of uninduced controls only 3 to 7 days after dox withdrawal [36]. Western blot analysis further revealed that saturating amounts of Ras are already reached with much lower concentrations of dox typically used to induce transgene expression. Hence, small amounts of dox can lead to a considerable increase in the protein expression level. In this case, a dox antagonist could be helpful to reduce or abrogate the effects caused by small, residual amounts of dox. This is also of considerable interest in the light of the increasingly sensitive third-generation rtTA variants which are up to 100-fold more sensitive towards dox than the first-generation, reaching effector response levels typical for tTA variants. With these novel reverse transregulators, already smallest amounts of dox would lead to target gene expression [37]. Taken together, dox withdrawal by medium exchange or exchange of drinking water is inefficient and only leads to a moderate and slow decrease in the expression level of the regulated gene due to poor dox clearance that can take days to weeks [23]–[25]. To demonstrate the advantage of K79 over this approach, we carried out transient transfection experiments in which either the dox-containing medium was removed or K79 was expressed in the presence of dox and luciferase activity was monitored after various time points (Figure 9). 10 h after exchanging the dox-containing medium with dox-free medium, luciferase activity decreased to about 50%. When GR_ΔNLS1_ was expressed, no significant change in luciferase activity was observed. However, when GR_ΔNLS1_-K79 was used, gene expression dropped to 15% and the response curve is characterized by a distinctly faster and stronger decrease in the expression level of the regulated gene. In the latter experiment, the dox-containing medium was not removed in samples in which GR_ΔNLS1_-K79 was expressed. Therefore, we next analyzed the extent of the decrease in target gene expression when both the dox-containing medium was exchanged with dox-free medium and GR_ΔNLS1_-K79 was expressed. As depicted in Figure 9B, this led to a 20-fold decrease in target gene expression after 10 h, equivalent to about 5% of the initial expression level.

However, the stability of the protein encoded by the regulated transgene has to be taken into account as well. For proteins with a long half-live, their stability could overbalance the effect of a rapid deactivation of rtTA by a dox antagonist. Therefore, the dox antagonist approach will be most efficient for genes encoding proteins with short to intermediate half-lives.

Hence, K79 not only represents the first rtTA-regulating peptide, it also provides the basis for a novel effector molecule. This peptide could serve as the starting point for a peptidomimetics-based approach to design a small molecule antagonist that could complement the widely used Tet-On system by delivering an efficient means to rapidly switch off rtTA-mediated activation of target gene expression at will.

## Materials and Methods

### Materials and general methods

Chemicals and antibodies were obtained from Merck, Sigma or Roth (Karlsruhe, Germany) and of the highest purity available. Media, buffers and solutions were prepared with deionized water and autoclaved. Heat-labile substances were dissolved and filtered with a 0.2 µm sterile filter. Enzymes for DNA restriction and modification were obtained from New England Biolabs, Roche Diagnostics, Invitrogen and Fermentas. Sequencing was carried out according to the protocol provided by PE Applied Biosystems for cycle sequencing.

### Bacterial strains, yeast strains and plasmids

The *E. coli* strains DH5α [38] and RB791 [39] were used for general cloning procedures. The *E. coli* strain WH207(λ*tet*50) [40] served as host strain for β-galactosidase assays and harbors a transposon Tn*10-*derived *tetA-lacZ* transcriptional fusion. The yeast strain AH109 was used for two-hybrid screening and served as host strain for β-galactosidase assays. Derivatives of the plasmids pWH1413, pWH527, pWH528 and pWH529 were used to express TetR [27] and pWH2101 derivatives for the expression of C-terminal TrxA-peptide fusions [41].

### Yeast-two hybrid *in vivo* selection

The plasmid pGBT9 was used for expressing the “bait” protein scTetR(B) fused to the GAL4 DNA binding domain (DBD). The plasmid pGADGH encodes the GAL4 activation domain (AD) fused to the Matchmaker™ Random Peptide Library (Clontech) which served as the “prey” protein. The yeast strain AH109 was transformed with pGBT9 and pGADGH and positive candidates were selected on minimal medium (Δhis, Δleu, Δtrp). The positive control consisted of the proteins Retinoblastoma, encoded by pGBT9-Rb, and phosphatase 1, encoded by pGADGH-P1. A detailed delineation of the experimental procedures is available upon request.

### Cloning of C-terminal TrxA-peptide fusions

Construction of the TrxA-peptide fusion proteins was done using standard PCR and cloning techniques. The peptide-encoding sequences were amplified from the respective pGADGH-peptide constructs and cloned into pWH2100 encoding TrxA [27]. The resulting constructs were designated pWH2100-*peptide* and verified by sequencing. For alanine scanning of K79, mutations were introduced using site-directed mutagenesis. Oligonucleotide sequences and detailed cloning procedures are available upon request.

### TetR variants used in this study

The *in vivo* selection was done using the reverse TetR variant TetR(B)S12G-E19G-A56P-D148E-H179R, the TetR moiety of rtTA2^S^-M2. For the *in vivo* analysis in *E. coli*, rtTA2^S^-M2 was expressed from pWH1413 mediating constitutive medium-level expression of TetR [27]. TetR(B) wildtype, TetR(BD) wildtype and tc-induction-deficient TetR(B) mutants [42] were encoded by pWH527 derivatives mediating constitutive low-level expression of TetR [27].

### β-galactosidase activity measurements in *E. coli*

Repression, inducibility, and co-repression of TetR was analyzed in *E. coli* WH207(λ*tet*50). This strain was co-transformed either with pWH527 or with pWH1413 derivatives expressing TetR, and with pWH2101 derivatives encoding the respective C-terminal TrxA peptide fusion. Overnight cultures and log-phase cultures were grown at 37°C in LB medium supplemented with the appropriate antibiotics. For this purpose, stationary phase cultures were diluted 1∶75 in fresh medium and expression of the fusion proteins was induced using 60 µM IPTG. The cells were then grown to an OD_600_ of approximately 0.4 and their β-galactosidase activities determined as described [29]. Three independent clones were analyzed for each combination of constructs and experiments repeated at least three times. The values obtained were normalized to the maximal β-galactosidase activity in the absence of TetR which was set to 100% and typically varied from 6500 to 7500 Miller units in the individual experiments. The data are shown as mean values of three independent biological samples in triplicate ± standard deviation.

### Western blot analysis

*E. coli* WH207(λ*tet*50) was transformed with TrxA- or TrxA-K79-expressing plasmids and overnight cultures were used to inoculate log-phase cultures (100 ml LB medium, 1∶100) which were grown to an OD_600_ of ≈0.5. Cells were harvested and resuspended in 500 µl PBS buffer supplemented with Complete™ protease inhibitor mixture (Roche) according to the manufacturer's protocol. Protein lysates were obtained by sonication and centrifugation at 13000 rpm, 4°C for 30 min. Proteins were separated using 15% SDS-PAGE and the gel was blotted overnight on a polyvinylidene difluoride membrane. As primary antibodies, a polyclonal anti-TrxA antibody (anti-Thio, Sigma) was used to detect TrxA and TrxA-K79, and a monoclonal anti-DnaK antibody (anti-DnaK (8E2/2), Enzo Life Sciences) was used to detect DnaK which served as loading control. Detection was done using the ECL kit according to the manufacturer's protocol (GE Healthcare).

### Transient transfections of HeLa cells

The HeLa cell line HLF33 [33] was used and cultured in Dulbecco's modified Eagle's medium (DMEM) without Phenolred (Gibco) supplemented with 10% charcoal-stripped fetal bovine serum (FBS), 100 µg/ml penicillin and 100 µg/ml streptomycin. Briefly, the charcoal-stripped FBS was obtained by suspending 10 g Norit A in 200 ml TRIS buffer (1 M, pH 7.4) and shaking for 4 h at 4°C. After addition of 100 mg dextran and shaking for 20 min at 4°C, activated charcoal was obtained by centrifugation for 10 min at 4000 rpm. Heat-inactivated FBS was added to the charcoal pellet and incubated for 3 h at 4°C. After centrifugation (4000 rpm, 10 min, 4°C), the supernatant was filter-sterilized and the charcoal-stripped, sterile FBS stored at −20°C.

3×10^4^ cells/well were seeded in 24-well plates for transient transfection assays which were carried out with Fugene HD ™ (Promega) according to the manufacturer's instructions. 10 ng of the rtTA-encoding plasmid and 100 ng of the GR-encoding plasmids were used and DNA mixtures were adjusted to a total of 250 ng DNA by adding non-specific pWHE121 plasmid DNA [43]. Doxycycline (dox) and dexamethasone were used at 2 µM and cells were harvested after approximately 24 h (see below).

Three wells were transfected in parallel per experiment, and experiments were performed at least three times. Cells were lysed approximately 20 h after effector addition by incubation with 100 µl lysis buffer [25 mM potassium phosphate (pH 7.8), 2 mM EDTA (pH 8.0), 5% glycerol, 1% Triton X-100, 20 mM dithiothreitol]. Firefly luciferase activity was determined with 10 µl cell lysate and a luciferase buffer [100 mM potassium phosphate (pH 7.8), 15 mM MgSO_4_, 5 mM ATP, 0.125 mM D-luciferin] using a Berthold Orion II luminometer. Light units were normalized for protein amount which was determined by a BioRad protein assay. The data are shown as mean values of three independent biological samples in triplicate ± standard deviation.

### Transient transfections of HeLa cells (time-dependence experiment)

For the transient transfections, the cells were treated as described above. Before preparing the crude lysates to determine luciferase activity, the cells were treated as follows (2, 4, 6, 8 and 10 h before lysis):

i) cells transfected with an rtTA-encoding plasmid: the dox-containing medium was removed and replaced with fresh medium without dox.

ii) cells transfected with an rtTA-encoding plasmid and a GR-K79-encoding plasmid: at time points 2, 4, 6, 8 and 10 h, dexamethasone was added to a final concentration of 2 µM to the dox-containing medium.
